# Consumer perceptions of beef healthiness: results from a qualitative study in four European countries

**DOI:** 10.1186/1471-2458-10-342

**Published:** 2010-06-15

**Authors:** Lynn Van Wezemael, Wim Verbeke, Marcia D de Barcellos, Joachim Scholderer, Federico Perez-Cueto

**Affiliations:** 1 Department of Agricultural Economics, Ghent University, Coupure Links 653, B-9000 Ghent, Belgium; 2 MAPP Centre for Research on Customer Relations in the Food Sector, Aarhus University, Haslegaardsvej 10, 8210 Aarhus, Denmark; 3 Post Graduate Programme in Business Administration (PPGAd), Pontifical Catholic University of Rio Grande do Sul (PUCRS), Av. Ipiranga 6681, Building 50, 1102, 90619-900, Porto Alegre, RS, Brazil

## Abstract

**Background:**

Consumer perception of the healthiness of beef is an important determinant of beef consumption. However, little is known about how consumers perceive the healthiness of beef. The aim of this study is to shed light on the associations between beef and health.

**Methods:**

Eight focus group discussions were conducted in four European countries (France, UK, Germany, Spain), each consisting of seven to nine participants. A content analysis was performed on the transcripts of these discussions.

**Results:**

Although beef was generally perceived as healthful, focus group participants expected positive as well as negative effects of beef consumption on their health. Labelled, branded, fresh and lean beef were perceived as signalling healthful beef, in contrast with further processed and packaged beef. Consumers felt that their individual choices could make a difference with respect to the healthiness of beef consumed. Focus group participants were not in favour of improving beef healthiness during processing, but rather focussed on appropriate consumption behaviour and preparation methods.

**Conclusions:**

The individual responsibility for health implies that consumers should be able to make correct judgements about how healthful their food is. However, the results of this study indicate that an accurate assessment of beef healthiness is not always straightforward. The presented results on consumer perceptions of beef healthiness provide insights into consumer decision making processes, which are important for the innovation and product differentiation in the European beef sector, as well as for public health policy decisions related to meat consumption in general and beef consumption in particular.

## Background

Although beef constitutes an important part of many consumers' diets, its consumption has become a quite controversial issue. On the one hand, red meat provides essential nutrients, containing high quality protein and essential micronutrients such as vitamins A, B_6_, B_12_, D and E, iron, zinc and selenium, contributing to consumers' health throughout life [1,2]. Therefore, the nutritional value has been key to communicate the health benefits of red meat to consumers [3]. On the other hand, over the last two decades, this positive image of the nutritional value of red meat has often been overshadowed by diverging developments in the consumer market and in the meat sector itself [2]. Consumers have become increasingly concerned about food-borne risks and personal health. As a consequence, consumer demand for safe and healthful foods has been increasing. The fat content and the possibly negative effect of red meat on consumers' cholesterol levels have become one of their major health concerns [4-6]. Also changes in consumer taste and preference have occurred, such as the increased consumption of processed meat products [7]. Furthermore, consumers have been increasingly expressing ethical and environmental concerns related to meat consumption, since beef production is particularly resource intensive and inefficient, putting pressure on the natural environment, climate, energy, water and biodiversity [5,8,9].

A main factor in the controversial nature of meat is the occurrence of food safety incidents. The meat sector, and especially the beef sector, is susceptible to food scares such as the BSE crisis, or the presence of harmful residues (e.g. dioxins) in the final products. These incidents, and the initial lack of responsiveness of the beef sector, have harmed the reputation of the sector [10]. Another important factor is the recent research and consumer interest in the association between red meat and cancer [2]. Altogether, the controversial nature of beef has been revealed by declining consumption rates and consumer confidence in developed countries [4,10] and has complicated a balanced judgement of the healthiness of beef by consumers [11]. Consumers may feel confused by receiving diverging and possibly contradicting information about the healthiness of beef. Therefore, despite conclusive evidence about the positive effect of the nutrients in beef when consumed in reasonable amounts as part of a varied diet, consumer perceptions of the healthiness of beef might not be univocally positive.

The perception of the healthiness of foods is influenced by various factors, such as type and processing of raw materials, origin, production date, preservation method, packaging and use of additives [12]. Consumers can only evaluate the nutritional content of the food by relying on nutritional labelling. The use of such information is higher for consumers who are more health-minded and consume beef less frequently [13]. The use of health claims on food products can enhance consumer perceptions of the healthiness of products [12,14]. From previous research it is known that health and nutrition considerations, such as cholesterol and saturated fat content, can play a role in consumer choices [13,15]. Therefore, it is important to know how consumers perceive the healthiness of beef. This knowledge is crucial in determining consumers' acceptance of new beef products to be developed [16] and in facing the challenges related to the current economic crisis. Most studies about consumer perception of beef have been performed in the aftermath of the BSE crises, focussing mostly on safety aspects. Little is known about current perceptions of beef healthiness [17]. Many intermediary factors that may influence beliefs about beef remain unknown, urging for more research in this field [11].

A common framework for the analysis of consumer quality perception and decision-making in the food sector is the Total Food Quality Model [18]. The model distinguishes between before and after purchase evaluations. In making food choices, consumers develop expectations about the quality characteristics of food products, including healthiness. Since healthiness is a credence attribute, consumers can only to a limited extent experience the real healthiness of the beef, even after purchase and consumption. The level of the healthiness of beef is neither clearly observable for consumers, nor can it readily be experienced. Consumers should have faith in the product, or rely on the available information, such as health claims. Because of this credence nature, this analysis focuses on the before purchase evaluation of the healthiness of beef. Before purchasing, expectations on the healthiness of beef are formed based on cues and information that are available for consumers [18], being intrinsic (physical characteristics of the product) or extrinsic cues. After the purchase, consumers might hardly ever experience the healthiness of beef, making it hard to compare the expected with the experienced healthiness.

In this paper, European consumers' perceptions of the healthiness of beef are explored. A qualitative study was conducted in four European countries, assessing consumer perceptions of the healthiness of beef. This research has been conducted as a part of the EU-funded project ProSafeBeef, aiming at innovation and diversification in the European beef sector. This paper adds to our knowledge and understanding of consumer perceptions related to beef by investigating expected health effects, the perceived role of beef in the diet, cues signalling (un)healthful beef and consumers' suggestions to improve the healthiness of beef. The results provide more insight into consumer decision making processes by exploring how consumers perceive and assess the healthiness of beef.

## Methods

### Focus group participants

During May 2008, focus group discussions were conducted in the capital cities of four European countries (Germany, Spain, France and the United Kingdom), which were selected because of their significant beef market volume and strategic geographical location within the EU. In each country, two group discussions were performed with each seven to nine participants. In total, 65 individuals participated in the study. To create more integration among the participants and less interference due to gender differences, focus groups did not mix men and women [19]. The number of participants in each focus group was determined based on general guidelines for conducting focus group research. Specifically, to facilitate and optimize conversation and discussion between the participants, it is recommended to select between six and eight participants [20]. The number of focus groups was determined based on practical and saturation criteria. Although four different countries were involved, the structured nature of the interview and the fact that the focus groups were not mixed in gender facilitated the achievement of saturation. After having conducted eight focus groups with the same structure and topic guide, additional data collection would no longer yield new insights or understandings about beef healthiness beliefs. All participants were beef eaters (defined in this study as consuming beef at least once a week) and beef shoppers without aversion to beef. Participants had a wide range of employment statuses and their age ranged from 19 to 60 years. Both individuals with and without children were participating in the focus group discussions, each covering around 2.5 hours.

All relevant international guidelines and ethical standards relating to the collection of personal data from human beings have been abided. Participants in the focus group discussions were adult healthy volunteers. They were recruited by a professional market research agency (TNS Gallup) that abides the ICC/ESOMAR International Code on Market and Social Research regarding ethics in social sciences research [21]. Participants were informed about the scope of the study and provided their written informed consent and agreement to participate. The interviews were videotaped for the purpose of later transcription and were destroyed immediately thereafter. Transcriptions were fully anonymous and participants were non-identifiable for analysis. As a result of these precaution measures concerning the data collection and processing, no formal ethical approval had to be sought from a competent national ethical committee.

### Interview guide

In accordance with the objectives of the overall consumer study of the ProSafeBeef project, focus group participants discussed perceptions and interest in beef safety, beef healthiness and related information, besides attitudes towards beef processing technologies [22,23]. The findings from the section of the group discussions which was concerned with beef healthiness are discussed in this paper. After discussing general beliefs about beef, specific beef healthiness issues were considered (including perceptions, cues and responsibility). For each focus group, the translated interview guide was applied to the participants in their respective languages.

### Data analysis

All eight focus group discussions were carefully transcribed. The full transcripts of the discussions in the local languages (covering more than 700 pages) were used as input for the content analysis, which was performed with the qualitative research software tool NVIVO7. Content analysis is a qualitative research tool to study the content of communication. This systematic and descriptive method is used to analyse words or phrases within a wider range of spoken or written communication. Strict procedures were followed to control for reflexivity bias. The first task was to create a code list with a common understanding across researchers. Inter-coder reliability was assured by a very intense collaboration during the development of the list of codes. Researchers iteratively compared coding decisions and discussed about the content of the codes, resulting in a list of codes which was consistently interpreted across researches. After agreeing on the code list, the transcripts of the focus groups were coded: the text was broken down into manageable categories of phrases and sentences, and labelled with the code(s) that reflected the content of these phrases and sentences. In that way, after coding all transcripts, a code contains all available information and statements about one concept. These can then be examined in detail to detect, for instance, trends, relationships with other concepts or conflicting aspects. Findings are reported including verbatim statements to illustrate the opinions and beliefs as reflected by the participants.

### Methodological limitations

The limited number of participants and the lack of representativeness imply that the results cannot be readily extrapolated to the population. This is not the objective of this exploratory research, though. Furthermore, the relative importance of the different concerns about beef healthiness is not reflected in the results, since focus group results do not allow sorting out the importance of the consumer opinions. While interpreting the results, these limitations should be kept in mind. Notwithstanding the methodological limitations, focus group studies have been proven to be valuable and were successful in exploring consumer perceptions in the domain of food-related behaviour [24].

## Results

**Figure 1:**
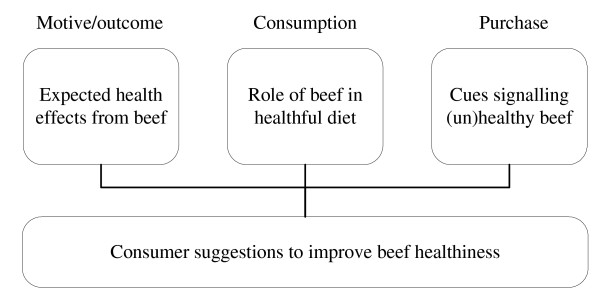
**Overview of the results from the focus group discussions**.

Health was important for all participants of the focus groups: "*Your health is the most important in life, and you must take care of that in any case*" (German woman, 28 years). Participants related health to being healthy and in good shape, well-being and happiness, power and sport, and a long and joyful life. The specific themes with respect to beef healthiness are discussed as structured in Figure 1.

### Expected positive health effects of consuming beef

Overall, most of the participants considered beef as healthful. First, consumers expressed high trust in food regulations: "*There are so many laws about everything that I would be surprised if they would get away with stuff that actually affects your health in a negative way*" (British man, 33 years).

Second, the nutritional value of beef was well recognized in all focus groups: "*Beef is nutritious*" (German woman, 48 years). The main focus was on iron and proteins, although they felt quite unsure about particular nutrients in beef: "*I think it can provide iron, when you are feeling low*" (British woman, 34 years), and "*beef has a good amount of proteins*" (German man, 41 years). Furthermore, beef was considered as a rather lean type of meat by most participants: "*Beef is healthful in the sense that it is pure, it is not fatty*" (British woman, 29 years) and "*it has less fat than other meats*" (Spanish woman, 48 years). Others disagreed: "*Its fat content is quite high, isn't it though?*" (British man, 44 years).

Because of its high nutritional value, beef was believed to "*provide strength, energy and vitality*" (French woman, 44 years), "*for people that work hard*" (British woman, 29 years). Multiple statements clearly expressed this belief: "*My children are of the sportive type and they need and eat a lot of beef*" (Spanish woman, 48 years), "*A meal has to give you power, you have to eat red meat*" (French man, 49 years) and "*Body-builders eat steaks as well, you know, to build themselves up*" (British man, 30 years). Although most consumers considered beef as indispensable in a healthful diet ("*I believe vegetarians have nutritional deficiencies*", Spanish woman, 48 years), some participants expressed doubts about the necessity of beef in the diet: "*I have vegetarian friends who do not eat beef and have not eaten it for years, so I do not think it is important in the nutritional sense*" (British woman, 34 years). In contrast, general agreement existed about the importance of beef in children's diets: "*For children, beef is a necessity, otherwise they will have deficiencies of iron and proteins. Children who do not consume meat, have major health problems*" (French women, 43 and 58 years). Furthermore, beef was stated to be good for bone formation and dental development, though for other perceived reasons than its vitamin and mineral content: "*It is still one of the meat types which allow us to chew*" (French man, 43 years).

### Expected negative health effects of consuming beef

Most consumers did not worry about beef healthiness: "*Meat is one of the last things I worry about, because you are conditioned to think it is good for you*" (British man, 21 years). Nevertheless, a number of consumers had some doubts: "*I would not say that beef in itself is healthful*" (German man, 41 years); "*It is not really healthful in a way because it is red meat*" (British woman, 37 years). Consumers identified various possible negative effects on human health. Concerns were expressed about the carcinogenic effect of beef consumption: "*I have read that there is a correlation between the amount of beef consumed and the growing number of cancers*" (French man, 20 years) and "*Baking and grilling, well, any change in the surface of the food can cause cancer*" (British women, 34 and 38 years). Concerns particularly related to the adverse accumulative effect of meat consumption in the long term: "*These diseases... it is not today, it will be in twenty years. Cancers and things like that*" (French woman, 44 years). Besides the expected carcinogenic effect, beef consumption was perceived as having "*negative outcomes for the veins*" (French man, 58 years) and "*increasing cholesterol levels*" (British woman, 37 years) and thus "*causing cardiovascular diseases*" (French man, 20 years). Another perceived potential danger was the transfer of animal diseases to humans: "*It causes Creutzfeldt-Jakob disease*" (French man, 58 years). Beef consumption could also promote obesity as perceived by some focus group participants, since "*while processing beef, they put a lot of fat in it*" (French woman, 20 years). According to some consumers, beef consumption might decrease life expectancy and cause death: "*We know that bad meat can kill*" (British woman, 34 years).

These negative effects of beef consumption on human health were perceived as related to the amount and type of beef consumed, the preparation method, and the presence of harmful residues in the beef. In the first place, the amount of beef was considered as important: "*Beef is healthful when consumed in the right amount*" (German man, 48 years). Beef was considered as harmful for human health at high consumption levels: "*Too much red meat can be bad for you*" (British man, 31 years). The participants agreed that moderation in the frequency of consumption is important: "*More than once but definitely not every day of the week*" (British man, 31 years), since "*anything in excess is not good for you*" (British woman, 34 years); "*I eat beef with moderation since it is unhealthful to eat too much meat*" (Spanish man, 26 years). Numerous statements in all focus groups suggested general support for moderate beef consumption, e.g. "*Everything depends on the way and in which amount the beef is consumed. Don't eat beef in excess*" (French man, 43 years). Not only the amount of beef consumed raised concerns, but also the best possible specific beef product and the preparation method ("*Some beef cuts are leaner than others*", French man, 43 years; "*The way you prepare it determines whether beef is healthful*", German man, 48 years). Consumers mentioned different preparation methods in this respect: "*After all it depends on how it is prepared. Whether you prepare your meat with a lot of butter or not*" (French man, 43 years); "*If you prepare it in a deep fat fryer, then you know that it is not very healthful*" (British woman, 40 years); and "*Steak or roast is better for your health than beef prepared with a sauce*" (French woman, 60 years). Interestingly, the "unhealthful" aspects were not directly related to beef as the core product, but rather related to the "side" and preparation ingredients such as butter, margarine, oil or sauce.

Focus group participants were also concerned about the presence of harmful residues in beef: "*I think beef is not intrinsically or naturally unhealthful, apart from the things that are inside*" (German man, 51 years). These residues might originate from animal feeding ("*What they are eating, in the fields, that is what caused BSE wasn't it?*", British man, 30 years) or from medicinal treatments or the use of growth hormones ("*Steroids are passing into the food chain*", British man, 30 years). Nevertheless, medical treatments were not experienced as exclusively detrimental: "*Vaccination of the animals to avoid illness is a good thing*" (French woman, 44 years).

### Perception of beef as a component of a healthful diet

A healthful diet is a balanced diet. That was the firm belief of all participants of the focus groups: "*The mixture of things you eat determines whether you eat healthful*" (German woman, 27 years). The right amount of beef was considered as an important part of a balanced diet: "*I try to achieve the right balance between vegetables and meat*" (French man, 24 years) and "*It is always important how much beef you eat and whether you have a balanced diet, with vegetables and fruit and other things*" (German man, 51 years). Discussing healthful diets, consumers also mentioned the importance of a low intake of calories and fat. Beef was perceived as a food product matching these recommendations: "*When you are careful with calories, you better eat beef*" (German woman, 47 years) and "*beef is good for a diet without too much fat*" (French woman, 60 years). Overall, beef was perceived as an important part of a balanced, healthful diet ("*Mediterranean diet is full of meat and stews*", Spanish man, 38 years), corresponding to a healthful lifestyle: "*It is the overall picture that is important: whether you do not drink alcohol, whether you do not smoke, whether you exercise*" (French man, 43 years).

Most participants assigned an equally important position in their meal to beef as to other types of meat, "*in competition with chicken and pork*" (French man, 35 years). While for some consumers beef was the main component of their diet throughout the lifecycle ("*We eat a lot more meat than fruits and vegetables*", French man, 51 years and "*All your life your mother has served filets*", Spanish man, 25 years), others put it into perspective: "*Beef is only one element of our food. In general, meat is a part of our food, but it is not the only thing we eat*" (French man, 43 years). Consumers stated that beef is more healthful than pork but less healthful than white meat. This idea was expressed in most focus groups, although the participants stated to experience lack of objective knowledge about this: "*I don't know why we think it, we just do generally think that beef is nutritionally better meat*" (British man, 21 years). Comparing beef to pork, "*beef is a more healthful choice*" (German woman, 28 years), since "*pork has a much higher fat content*" (British man, 30 years). Beef was perceived as less healthful than white meat: "*I think red meat is not as good for you as poultry or turkey*" (British woman, 41 years), since "*white meat is more nutritious and healthful and has less calories*" (German woman, 28 years) and "*beef has much more fat than white meat*" (French man, 24 years). The male French participants also mentioned horsemeat, which was considered as more healthful than beef since "*beef is more fat than horsemeat*" (French man, 58 years), and "*horsemeat is better from a nutritional point of view*" (French man, 24 years).

### Cues signalling (un)healthful beef

To assess beef healthiness, consumers indicated to use specific characteristics of beef as beef healthiness cues. Both intrinsic and extrinsic healthiness cues are used. Table 1 shows which product or processing characteristics and categories of beef were perceived as healthful versus unhealthful by the participants of the focus groups. The participants of the focus groups did not reach a consensus whether organic beef was healthful or unhealthful. Some consumers believed organic beef to be healthful: "*Organic beef is good for your health because the animals are fed naturally without any additives*" (French woman, 28 years), while others stated: "*Health wise there is really no difference between organic and ordinary beef*" (British woman, 40 years).

**Table 1:** Cues signalling healthful and unhealthful beef

Healthful beef	Unhealthful beef
Labelled beef	BSE/food scares
Branded beef	Ready meals
Lean beef	Expired beef
Good appearance	Offals
Fresh beef	Sold and processed in unhygienic conditions
'Natural' beef (well fed, well treated, only slightly processed)	Packaged beef
Properly cooked beef	Canned beef
Beef from big vendors	Further processed beef products
Beef in a balanced diet	Low quality beef
Organic beef*	Beef with additives
	Beef with hormones
	Cheap beef
	Organic beef*

* No consensus

### Consumers' suggestions to improve the healthiness of beef

The participants of the focus groups suggested that changing the methods of production could make beef more healthful: "*Beef healthiness is related to the breeding practices. Maybe we need to create or enforce the rules for cattle breeding*" (French man, 30 years). The whole process should be taken into account: "*from slaughter to processing including the addition of all kinds of additives and preservatives*" (Spanish man, 37 years). Producing more healthful beef was in the first place associated with appropriate feeding: "*Beef can become more healthful with natural feeding*" (French man, 51 years). The cows should get "*a natural diet, what cows naturally eat, grass*" (British woman, 29 years), instead of "being fed with chemicals" (German man, 29 years). The calf should be allowed to drink milk instead of "*being fed with powder*" (French man, 34 years) and "*the animals should not be fattened*" (German man, 41 years). Therefore, some consumers suggested applying organic methods of production, in which "*the animals are raised with natural products without additives, without chemical products added*" (French woman, 28 years). Besides the feeding of the animals, consumers recommended appropriate cattle rearing ("*Cows should be kept very naturally, on grass fields, in prairies. Not in a small place*", German man, 29 years), a correct treatment of the animals ("*A stressed animal can have diseases that are not even known to man, caused by the maltreatment of the animal*", French woman, 28 years), and serene methods of slaughtering ("*Living naturally, killing humanely, then it is healthful beef*", French man, 49 years).

The idea of improving the healthiness of beef during processing was not unconditionally accepted by all participants. Doubts were expressed whether the manipulation of beef would indeed make it more healthful ("*More healthful? More healthful than leaving the meat alone and just being a good cook?*", British woman, 34 years) and whether it would mean a real improvement ("*What are they going to do to make beef more lean? If I have fat on my beef I know it is normal*", British women, 37 and 40 years). Furthermore, uncertainty existed about short term health effects ("*By continuously improving the healthiness and safety of our food, we might suppress the natural defence system of our own body*", French man, 43 years) as well as long term effects ("*It is meant to improve the healthiness of beef, but today they add some additives and they don't know whether it will cause diseases tomorrow*", French woman, 28 years). Moreover, addition of healthy compounds such as omega-3 fatty acids was thought to "*compromise taste*" (Spanish man, 38 years).

While the participants of the focus groups were not unconditionally in favour of improving beef healthiness by the manipulation of beef, they did suggest that an altered consumption of beef might mean an improvement in terms of health. Since beef was considered as healthful in itself, it was rather a matter of making healthful consumer choices and adapting habits and behaviours: "*The more healthful beef is there. If you choose not to have it, if you do not put it in your basket, then it is not there*" (British man, 35 years). Since "*beef cuts which are not or only slightly processed are more healthful*" (German woman, 45 years), it was stated that it was a matter of "*choosing the right beef cut*" (French man, 43 years). The same applied to the methods of preparation. Appropriate methods of preparation could also lead to more healthful beef products. The method of cooking was perceived as a decisive factor ("*It is likely that viruses and bacteria are killed, in beef products that are cooked or heated for a longer time*", German man, 51 years). Interestingly, possible adverse health effects from cooking beef overdone were not mentioned. The participants suggested that consumer choice and methods of preparation are often "*determined by someone's upbringing*" (French woman, 43 years).

## Discussion

This paper provides exploratory qualitative results from focus group discussions. The limited number of participants, the lack of representativeness and the exploratory nature of the study imply that the results cannot be readily extrapolated to the overall population. To avoid overgeneralisation of the findings, the interpretation of focus group results requires care. This limitation should be kept in mind while discussing the results.

### Consumer perceptions of the healthiness of beef

The objective of this paper is to explore how consumers perceive and assess the healthiness of beef. The results show that in general, most of the participants of the focus groups considered beef to be a healthful food product. Since all participants were beef eaters, it is possible that this belief was stated as an ex post justification of their consumption of beef, which is consistent with the concept of post rationalisation. Both positive and negative expected health consequences of beef consumption were expressed by the focus group participants. Consumers make a trade-off between the risks and benefits of beef consumption: the expected negative health effects are not only balanced against the positive health effects, but also other factors are taken into account, such as taste and convenience [7,10].

The focus group participants listed several factors that are perceived to influence the healthiness of beef: the consumption amount, the type of product or cut, the preparation method, and the presence of harmful residues. Remarkable is that three of these four factors come within the compass of consumers' own responsibility. Because of the typical credence nature of the issue, the presence of harmful residues cannot be reduced by adapting individual consumers' choices while purchasing, preparing or consuming beef or beef products. This finding indicates that consumers recognize that their own consumption decisions have an important impact on their health and that they are (at least partly) responsible for the healthiness of their food. This view has been well documented in literature. Since the late 1970 s, health issues have become moral questions, with healthful diets representing proper moral behaviour, making individuals responsible for their personal health and lifestyle changes. This phenomenon has been called 'healthization' [5,25].

While the importance of the consumption frequency and quantity for the overall perceived healthiness of beef was clear for most focus group participants, the "most healthful" level of beef consumption was not. Moderation in consumption was highlighted, fitting the idea of a balanced diet and avoiding a too high level of red meat consumption, which has been linked to negative health consequences like cancer and heart diseases [9,26]. None of the focus group participants indicated the need to diminish their beef consumption: they talked about moderation without judging their own consumption behaviour. The observed confusion about the most healthful level of beef consumption is consistent with the results of a recent study, showing that consumers were not sure about whether or not they have to diminish their red meat consumption [27]. However, previous research suggested that many consumers believed that they should diminish their consumption of red meat [17] and effectively intended to do so [14]. This belief is subject to regional differences: Martinez-Gonzalez et al. [28] found that in Mediterranean countries, the notion of diminishing red meat consumption was more often found to be part of the perception of a healthful diet, compared to central and northern European countries. Expert opinions indeed indicate that consumers should limit the consumption of (especially processed) red meat [9]. However, the reports on the health risks of meat consumption are controversial. The risk might not be a function of meat per se, but reflect a high-fat intake and/or the generation of carcinogens through cooking and processing [29].

Participants evaluated the healthiness of meat in terms of fat, calories and nutritional value. Using these criteria, beef was deemed more healthful than pork, but less healthful than poultry meat or horsemeat. This ranking in consumer perception of meat attributes is consistent over time and did not even change over the dioxin crisis [6,30]. The focus group participants were concerned by the presence of harmful residues in beef, as well as by food scares such as BSE. Yet it has been substantiated that nutrition related risks are more relevant with respect to chronic diseases than with respect to the presence of residues or the occurrence of zoonosis [31]. Therefore, the presence of harmful residues in beef might be perceived by consumers as a larger risk than it is actually. Grunert [32] argued that food safety perceptions can act as "sleeping giants": under normal circumstances they do not influence quality perceptions, but in times of crisis they can have far-reaching effects.

### How consumers form expectations about the healthiness of beef

The Total Food Quality Model shows how expectations are formed by consumers based on cues that are available in the shopping situation. In line with previous research [18] the focus group participants listed both intrinsic cues (such as cut, appearance and fat content), and extrinsic cues (such as packaging, brand, labels and price) to assess beef healthiness prior to and during purchase (see Table 1). The participants also mentioned cues that were related to practices after the purchase (preparation and consumption), which are discussed further.

The cues listed in Table 1 reflect some general ideas about healthful eating. A healthful diet was defined as a balanced diet, containing a low amount of fat and calories. Moderate beef consumption was accepted as ingredient in a healthful diet. In accordance with previous reports, the participants described healthful eating habits focussing on balanced diets, fresh foods and natural or unprocessed foods [17,27]. This was also illustrated by the participants' perception of natural beef as more healthful than processed beef products and ready meals.

The participants of the focus groups stated that beef is lean meat and therefore healthful. The belief that fat is bad for health (though good for taste) corresponds with previous findings that food high in fat is perceived as unhealthful [33,34]. In fact, meat healthiness is largely related to its fat content and its fatty acids composition [35]. The participants of the focus groups did not differentiate between saturated and unsaturated fat. Meat is, however, a significant source of dietary essential fatty acids, although the concentrations are lower than in oily fish [34].

While it is known that packaging has an impact on the quality perception of products [36], the effect of packaging on healthiness perceptions has not been described in literature. Several focus group participants indicated that packaged beef was perceived as unhealthful. This might be due to the decreased visibility of intrinsic cues, since appearance is an important cue for consumers [4]. Another possible explanation is that consumers do not perceive packaged products as fresh products, or that they might associate it with the use of additives. Further research is needed to assess the importance of the packaging in consumers' healthiness perceptions and associations. A possible approach to this situation might be to put brand labels or quality certificates on the packaging, since focus group participants qualified both labelled and branded beef as healthful. This suggests a considerable level of trust in labels and brands, corresponding with recent findings in literature that food with a brand, quality label or health claim might be perceived healthier by consumers [18,37], contrary to the situation during the second half of the nineties when meat labels were regarded as suspicious [38]. Nevertheless, the perceived healthiness of labelled food often lacks accuracy [39], and the credibility of health claims on labels differs significantly [40]. Possibly the higher perceived healthiness of labelled and branded beef is related to its higher perceived quality [18]. In contrast, since fresh meat is mostly unbranded and unlabelled, consumers have to base their healthiness assessment mostly on the appearance of the product [7].

The focus group participants did not agree on the healthiness of organic beef. Many previous consumer studies, however, have assessed consumer perceptions of organic foods, most of them concluding that organic food is perceived as safer and more healthful than conventionally produced food. Health and safety are even perceived as the most important quality attributes by organic food consumers [41]. Beef, compared to vegetables and fruits for example, is not commonly associated with an organic production method, which may explain the uncertainty among the focus group participants.

Figure 1 also lists some healthiness cues that are used after purchase. These cues are related to preparation (appropriate methods) and consumption practices (balanced food). This again indicates that consumers acknowledge that they have some own contribution in the healthiness of the beef they consume.

### Improvement of the healthiness of beef

Consumers' suggestions to improve the healthiness of beef relate to various phases in the beef supply chain. Participants were sceptical about the improvement of the healthiness of beef by applying different or advanced processing methods. Manipulation of beef is perceived negatively, since consumers like their food being 'natural'. The concept of improving healthiness during processing contradicts with consumers' perception of beef healthiness. If the healthiness of beef should be improved, consumers would prefer it to happen in the production phase of the beef chain. Currently, the beef sector tries to improve the healthiness of beef both in the production phase (for instance by adjusting the feed to influence the fatty acids composition of beef [2]) and the processing phase (for instance marinating to reduce the formation of carcinogenic compound during grilling [42]). Hence, the adding of potentially healthful and natural ingredients (such as olive oil and herb-based seasonings) in beef processing could increase the chances of acceptance of such products. The focus group participants indicated to be aware of their own responsibility and possible impact in the improvement of beef healthiness by their personal choices and consumption behaviour. Finally, based on participants' opinions, the catering and food service industry could benefit from the offer of healthful, ready-to-eat beef meals. The trend towards convenience is a reality and the consumption of "food on-the-go" and "take-away" is particularly true in the dawn of the new century. Since this trend is intrinsically connected with modern lifestyles, the challenge to the beef industry would be to lead a repositioning of "junk, fast food" into more healthful convenience beef options.

## Conclusions

This paper explores consumers' perception and assessment of beef healthiness. The results from eight focus group discussions in four European countries provided insights into the expected health consequences of beef consumption, the position of beef in the diet, cues signalling (un)healthful beef and consumers' suggestions to improve the healthiness of beef.

In general, beef was perceived as a healthful component of the diet. Focus group participants expected both positive and negative health consequences of beef consumption. Labelled, branded, fresh and lean beef were perceived as healthful, in contrast with further processed and packaged beef. An original finding from this study is that consumers believed that their individual choices can make a difference with respect to the healthiness of beef consumed. On the one hand, the awareness of individual responsibility for health suggests that food industries and retailers could benefit from the supply of healthful beef products to consumers. On the other hand, it implies that consumers should be enabled to make correct judgements about the healthiness of their food. However, the results of this study indicate that an accurate assessment of beef healthiness is not always straightforward and feasible for an individual consumer. Consumers use various cues to evaluate the healthiness of beef. Although these results corroborate previous findings on how consumers form expectations about the healthiness of beef (using both intrinsic and extrinsic cues), one of the main contributions of this study is the finding that participants were sceptical about the improvement of the healthiness of beef by applying unfamiliar or advanced processing methods. This knowledge is crucial in determining consumers' acceptance of new beef products and stimulating beef industry competitiveness. Finally, an interesting and original finding from our study is that participants did not agree on the healthiness of organic beef. Previous consumer studies found that organic food is perceived as more safe and healthful than conventionally produced food. We believe that beef, unlike vegetables and fruits, is not commonly associated with organic production methods, which may explain the uncertainty among the focus group participants.

International dietary recommendations systematically advocate for increased consumption of fruits and vegetables, a variety of foods, and limited consumption of meat, especially processed meat products. Consumers are faced with conflicting messages about whether food products may be healthful or not (such as the association of some foods with cancer risk or the prevention of chronic diseases). Hence they have to develop easy and practical strategies and decision rules to make the best choices as the present study has shown. Therefore, clear messages through product information, labelling and advertising may facilitate consumer's product evaluation and decision making. The results of this study challenge producers to make healthful and convenient beef cuts available for the general population, as well as regulators to consider the interests of consumers and citizens. This way, achievement of public health goals could be facilitated.

The question remains whether consumers have the right impression of the health consequences, the factors determining whether beef is healthful or not, and the used information cues to infer beef healthiness. Perceptions are subjective notions because they reflect opinions about an objective reality. Although in fact such perceptions may be true or not, the individual is likely to act on these perceptions, hence creating real consequences (cf. the Thomas theorem) [43]. The presented results on consumer perceptions of beef healthiness provide insights into consumer decision making processes, which are important for the innovation and diversification in the European beef sector, as well as for public health policy decisions related to meat consumption in general and beef consumption in particular.

## Competing interests

The authors declare that they have no competing interests.

## Authors' contributions

LVW participated in the study design, performed the content analysis and drafted the manuscript. WV participated in the study design, revised the manuscript for important intellectual content and coordinated the project. MDB and FPC contributed to the content analysis and revised the manuscript for important intellectual content. JS participated in the study design and revised the manuscript for important intellectual content. All authors read and approved the final manuscript.

## Pre-publication history

The pre-publication history for this paper can be accessed here:

http://www.biomedcentral.com/1471-2458/10/342/prepub
